# Mesoplastics: A Review of Contamination Status, Analytical Methods, Pollution Sources, Potential Risks, and Future Perspectives of an Emerging Global Environmental Pollutant

**DOI:** 10.3390/toxics13030227

**Published:** 2025-03-20

**Authors:** Dioniela Mae C. Ellos, Mei-Fang Chien, Chihiro Inoue, Haruka Nakano, Atsuhiko Isobe, Deo Florence L. Onda, Kozo Watanabe, Hernando P. Bacosa

**Affiliations:** 1 Department of Environmental Science, School of Interdisciplinary Studies, Iligan Institute of Technology, Mindanao State University, Andres Bonifacio Avenue, Iligan 9200, Philippines; dionielamae.ellos@g.msuiit.edu.ph; 2 Graduate School of Environmental Studies, Tohoku University, Aramaki Aza Aoba 6-6-20 Aoba-ku, Sendai 980-8579, Japan; meifangchien@tohoku.ac.jp (M.-F.C.); chihiro.inoue.b1@tohoku.ac.jp (C.I.); 3 Research Institute for Applied Mechanics, Kyushu University, Kasuga 816-8580, Japan; nakano.hrk@riam.kyushu-u.ac.jp (H.N.); aisobe@riam.kyushu-u.ac.jp (A.I.); 4 Center for Ocean Plastic Studies, Kyushu University, CU Research Building 14th Floor, 254 Phaya Thai Rd, Wang Mai, Pathum Wan, Bangkok 10330, Thailand; 5 The Marine Science Institute, University of the Philippines, Velasquez Street, Diliman, Quezon City 1101, Philippines; dfonda@msi.upd.edu.ph; 6 Center for Marine Environmental Studies (CMES), Ehime University, Bunkyo-cho 3, Matsuyama 790-8577, Japan; watanabe.kozo.mj@ehime-u.ac.jp

**Keywords:** contamination, fragments, marine, polyethylene, pollution, polymer

## Abstract

Mesoplastics are emerging environmental pollutants that can pose a threat to the environment. Researching mesoplastics is crucial as they bridge the gap between macroplastics and microplastics by determining their role in plastic fragmentation and pathways, as well as their ecological impact. Investigating mesoplastic sources will help develop targeted policies and mitigation strategies to address plastic pollution. These pollutants are found across aquatic, terrestrial, and agricultural ecosystems. Unlike microplastics, mesoplastics are reviewed in the scientific literature. This paper focuses on existing published research on mesoplastics, determining the trends and synthesizing key findings related to mesoplastic pollution. Research primarily focused on marine and freshwater ecosystems, with surface water and beach sediments being the most studied compartments. Mesoplastics research often offers baseline data, with increased publications from 2014 to 2024, particularly in East Asia. However, certain ecosystems and regions remain underrepresented. Also, mesoplastics can disrupt ecosystems by degrading biodiversity, contaminating soils and waters, and affecting food chains. Mesoplastics can also become vectors for additives and pathogenic microorganisms, highlighting their environmental risks. Various factors influence mesoplastics’ prevalence, including anthropogenic and non-anthropogenic activities. With this, future research should expand into less-studied ecosystems and regions, explore mesoplastic interactions with pollutants and organisms, and promote public awareness, education, and policy measures to reduce plastic use and mitigate pollution globally.

## 1. Introduction

Plastic pollution is a current global issue since plastics are ubiquitous environmental contaminants. These materials persist in our environment, and >400 years from today, they could still remain in the environment [1], leading to widespread pollution in marine and terrestrial ecosystems and harming wildlife through ingestion and entanglement [2]. Additionally, plastics degrade into microplastics, which accumulate in food chains and may have toxic effects on organisms and human health. Plastics are highly favored raw materials for use in products by various sectors, especially the industrial sector, due to their low manufacturing cost, flexibility, lightweight, and longevity [3]. The global production of plastics has been increasing at a fast rate, with more than 360 million metric tons produced every year, with polyethylene, polypropylene, and polyvinyl chloride (PVC) being the most common plastics produced [2]. Polyethylene (PE) is a polymer produced by the polymerization of ethylene monomers into long chains of carbon atoms, making them very resistant to biodegradation and persistent in the environment [4]. In the same way, polypropylene (PP) is composed of propylene monomers, and its chemical structure with its methyl side groups confers it with greater crystallinity, hence making it rigid and resistant to degradation, which is environmentally problematic as it builds up in ecosystems [4]. Globally, plastic production has not shown signs of slowing down and is even predicted to hit 33 billion tons by 2050 [3]. The main problem with plastic goods is that their properties make them an environmental challenge, both from a physical and chemical perspective, as they are mostly made up of petroleum products [5], hence enriching the global fossil fuel supplies and ultimately contributing to climate change. Furthermore, plastics usually have numerous additives, such as plasticizers, stabilizers, flame retardants, and colorants, which improve their performance when used but present additional environmental risks. Additives from plastics can leach into ecosystems, leading to toxicity and concentration in the food chain [6]. Moreover, about 23 million metric tons of plastic are dumped and/or leaked into various aquatic ecosystems, especially the oceans, annually. In comparison, terrestrial ecosystems receive around 25 million metric tons of plastic each year [7].

Plastic pollution can be associated with plastics of different sizes, types, colors, and compositions, and has been reported to be found in various environmental systems [8,9,10]. The prevalence of plastic pollution has reached a critical stage, as it is now considered a hazard to both human well-being and the environment [11]. In terms of size, the most commonly identified classes are macroplastics (>25 mm), microplastics (<5 mm), and nanoplastics (1 nm to 1 mm), and among these, mesoplastics are the least discussed. Mesoplastics are formed from the breakdown of macroplastics and are larger compared to microplastics but smaller than macroplastics, with sizes ranging from 5 to 25 mm [12,13].

**Figure 1:**
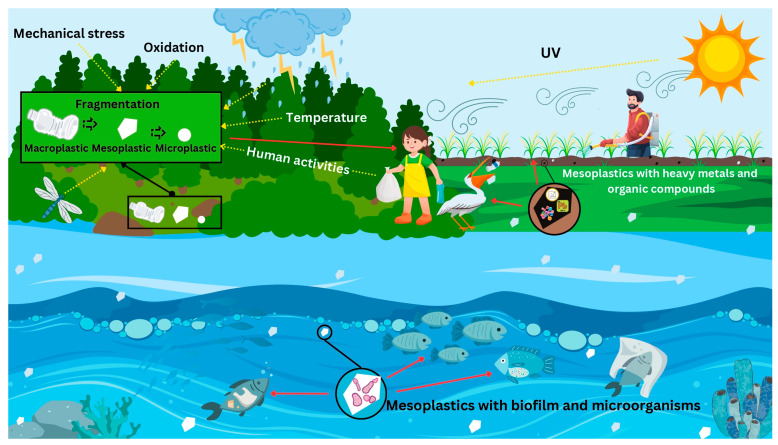
Schematic diagram of mesoplastics in the environment, the weathering factors, possible sources, and potential effects.

Mesoplastics, along with other plastic sizes, such as macro- and microplastics, enter the aquatic freshwater and marine ecosystems through different sources, mainly from land-based origins, including residential, tourism, and industrial activities, harbor operations, and even waste from wastewater treatment facilities [12] (Figure 1). With the expansive distribution of these plastics in the aquatic ecosystem, there is no doubt these materials can be consumed by organisms in some way. In a study conducted on the beaches of Istanbul, Turkey, a total of 3787 debris items were collected, with many plastics debris being categorized into macroplastics (47.8%) and mesoplastics (9.2%). All the beaches they studied could be classified as extremely dirty based on the Clean Coast index, with some of the plastic debris collected showing signs of extended deposition [14]. The beaches of the Laurentian Great Lakes of North America also exhibited plastic pollution, with the majority of the debris being large microplastics (68.4%) followed by mesoplastics (27.3%) and macroplastics (4.3%). Furthermore, pellets were the most abundant plastic type based on shape [15]. These results highlight that plastic pollution is increasingly prevalent in both marine and freshwater ecosystems.

Plastic pollution from agricultural land is also a serious issue. Out of the 265 million tons of plastic produced in 2010, 2% was used in the agricultural sector [16]. Plastics are commonly introduced to the soil/farmlands through plastic mulching. Plastic mulching is an agricultural practice common in the suburban agriculture area of Shanghai [17], in which the plastics covering the crops can degrade into much smaller debris [18]. China is estimated to consume about 60% of the global agricultural film on the market [19]. Moreover, agricultural lands in urban areas are expected to contain a large number of plastics, especially microplastics [20,21]. Aside from plastic mulching, plastics are commonly used in agricultural products, including covering films, shadings, nets, sacks, pots, etc., highlighting the wide range of uses of plastics [16]. In China, 93.1% of the collected micro/meso plastics(MMPs) are made up of polypropylene, polyethylene, and polyester and are mostly abundant in the arid to semi-arid northern part of China [22].

Most countries have yet to embrace the shift towards a circular economy. On a positive note, some countries are seeking cost-effective solutions to manage their plastic waste. These include post-consumption management in which infrastructure and capacity building are emphasized, reduction of plastic use by using more sustainable materials, and a ban on single-use plastics [23]. Furthermore, to gradually and steadily see positive changes regarding global plastic awareness, it is crucial to involve coordinated multi-stakeholder efforts from local to national levels [24].

Research on mesoplastics is crucial as they act as a bridge, filling the gap between macroplastics and microplastics, thereby contributing to our understanding of the lifecycle of plastic pollution. While there is an abundance of studies focused on marine and aquatic ecosystems and microplastics [21], the presence and distribution of mesoplastics across the various types of ecosystems remain underexplored and insufficiently reviewed. Investigating mesoplastics provides a more complete picture of how plastic debris transitions between different sizes, highlighting the continuous nature of plastic pollution in various ecosystems. While microplastics pose significant risks due to their widespread presence and ability to be ingested by smaller organisms, mesoplastics are more visible and can directly affect larger marine species, which can serve as an entry point for plastic into the food chain, which eventually break down further into microplastics, retaining the cycle of plastic pollution. Furthermore, studies on mesoplastics are particularly significant because they offer valuable insights into the plastic fragmentation process, showing how larger macroplastics degrade into smaller, more persistent particles over time. Due to their size, mesoplastics can act as carriers for toxic substances and pathogens, posing serious health and ecological risks. This review paper aims to determine the trends of the existing literature on mesoplastic pollution and analyze potential sources and risks, the chemicals associated with mesoplastics, the microorganisms that can inhabit mesoplastics, and the organisms that can aid in monitoring and degrading mesoplastics. By synthesizing the published articles on mesoplastic pollution through an integrative review, this work will provide a snapshot of the current research, highlight areas for future studies, and suggest studies that others may undertake. This research does not isolate mesoplastics but rather integrates them into the broader context of plastic waste accumulation, degradation, and distribution in the environment.

## 2. Methodology

Google Scholar was the primary site utilized to identify the appropriate articles that could be considered and synthesized for this review paper. For a broader literature search, Scopus, Web of Science, and PubMed were also used. The papers synthesized were those published from January 2014 to November 2024, and the papers were searched from August 2024 to November 2024. From there, several journals and publishers were visited, including Scopus-indexed and Web of Science-indexed journals. A total of 111 studies were considered for this review paper.

To ensure coherent flow of the paper, the topics are organized into the following sections: (1) trends on mesoplastics in the published research; (2) mesoplastics in the different ecosystems (i.e., marine, freshwater, terrestrial, agricultural); (3) phenomena affecting the prevalence of mesoplastics; (4) types of mesoplastics based on shape, color and polymer composition; (5) association of mesoplastics with macro, micro and nanoplastics; (6) analytical methods used for mesoplastic studies; (7) index tools that are used to determine mesoplastic contamination; (8) mesoplastics as vectors of inorganic and organic compounds; (9) mesoplastics affecting organisms; (10) mesoplastics as habitats of microorganisms; and, (11) utilization of various organisms for plastic monitoring and plastic degradation. In the search engine, the keyword “mesoplastics” was combined with “freshwater”, “marine”, “agricultural”, “terrestrial”, “factors”, “toxic chemicals”, “microorganisms”, “virus”, “bacteria”, “protist”, “organic compounds”, “index”, “monitoring”, and “degradation”. Duplicates and papers not related to the sections identified were discarded.

Data on the types of ecosystems where mesoplastics were collected, physical and chemical characterization of mesoplastics, collection methods, mesoplastic digestion and flotation techniques, identification techniques, the year of publication, unit of measurement used for mesoplastic concentration determination, the association between mesoplastics and macro, micro and nanoplastics, the trends of use of risk, and pollution indices were extracted with the use of Microsoft Excel.

## 3. Results and Discussion

### 3.1. Trends in Published Research on Mesoplastics

#### 3.1.1. Location of Study Area

Since this review paper focuses on a global setting, the frequency of publications (n = 109) on mesoplastics in different regions of the world was determined (Table 1). East Asia (15.6%) and South America and Southern Europe (10.09%) have the highest number of published articles on mesoplastic pollution. Next, there is a moderate number of published research in West Asia (7.34%), Southeast Asia (6.432%), North America (5.50%), South Asia (5.50%), and Southwestern Europe (5.50%). Australia, East Africa, Northeastern Africa, Northwest America, Oceania, and West Africa, with 0.91%, have almost no publications on the prevalence of mesoplastics. The global focus on mesoplastics in research is unbalanced; hence, it is essential to prioritize studying mesoplastics in continents that have not received much attention to gain a comprehensive understanding of their impact on the environment. Expanding research to under-studied regions can provide crucial insights into regional sources and distribution patterns.

#### 3.1.2. Year of Publication

**Figure 2:**
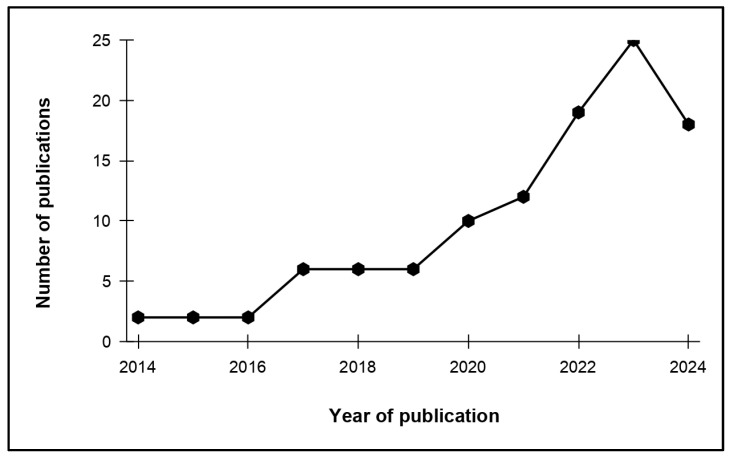
Number of journal articles published on the prevalence of mesoplastics in the past ten years (2014–2024).

A steadily increasing trend in the number of published works on mesoplastics was observed from 2014, peaking in 2023 (Figure 2). A drastic increase was observed between 2014 and 2018. There was also a noticeable increase in publications in 2019, suggesting growing global attention on mesoplastic pollution in the environment. This increase in research can be linked to the growing recognition of the environmental and health risks associated with plastic pollution, such as offering valuable insights into the plastic fragmentation process and demonstrating how larger macroplastics break down into smaller, more persistent particles over time. As the papers were analyzed up to September 2024, though it is very early to say if this is a pattern, the small reduction in 2024 might be due to stabilization or a change in the focus of research. Also, the patterns may be influenced by the timing of the review, as the studies were identified midway through the year.

### 3.2. Mesoplastics in Different Ecosystems

**Figure 3:**
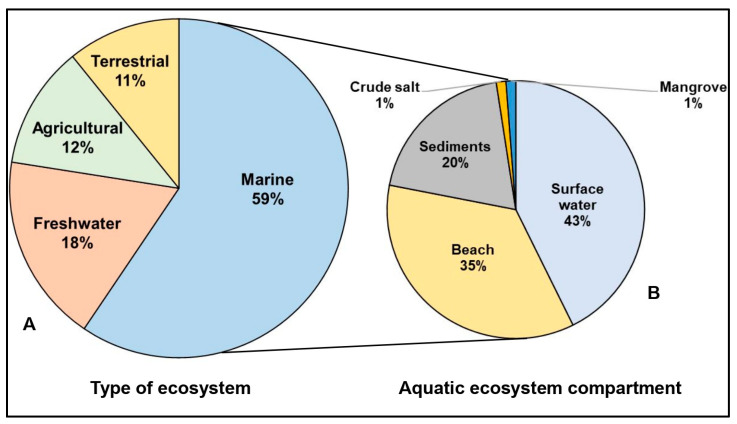
Number of published journal articles on mesoplastic pollution (**A**) across different types of ecosystems (n = 111) and (**B**) compartments of the aquatic ecosystem (n = 82).

This review paper determined that mesoplastics were detected in four types of ecosystems, namely marine waters, freshwater, terrestrial, and agricultural environments (Figure 3A), based on the published articles (n = 111). Of these four categories, the majority are studies in the marine or saltwater ecosystem (59.5%), followed by studies on mesoplastics in freshwater ecosystems (18.02%), agricultural areas (11.71%), and terrestrial ecosystems (10.81%). This trend suggests a strong research emphasis on mesoplastics in the aquatic ecosystem.

The distribution of journal articles (n = 82) on mesoplastic pollution across the various compartments of an aquatic ecosystem is shown in Figure 3B. Studies on mesoplastic pollution on surface waters and beaches had the highest number of publications, representing 42.68% and 35.37% of the reviewed studies, respectively. This trend suggests a strong accessibility of these ecosystems for sampling. In surface water plastic litter collection, the main method that can be utilized is the use of Manta nets, which consist of fine-mesh nets and buoyant wings, and Neuston, which are designed for continuous-flow sampling. This method often includes a flowmeter to measure seawater volume for accurate data collection [25]. Furthermore, studies showed a high abundance of mesoplastic pollution on beaches, which can also be associated with the high accumulation of marine debris, making them critical locations for monitoring plastic pollution and identifying its sources. Factors such as local water current patterns, weather conditions, exposure levels, and beach characteristics like substrate type, slope, and curvature may influence mesoplastic accumulation on beaches [26]. While some studies indicate that coastal and deep-sea sediments are major sinks for plastics, global estimates are limited due to the regional nature of plastic research [27]. Ref. [28] investigated the presence of mesoplastics in different segments of the Cox’s Bazar coast. They found that mesoplastics were more abundant in beach sediments compared to the surface water and crude salt compartments. The most commonly observed types of plastic polymers are polyethylene, polypropylene, and polystyrene [28]. In Gualí Wetland Cundinamarca, located in Colombia, mesoplastics were more abundant than both macro- and microplastics, with HDPE being the most abundant polymer type, mainly in the shapes of fragments and pellets [29]. In light of this, further research is needed on aquatic ecosystems that have not received much attention so far, such as mangrove forests, which may facilitate the accumulation and could thus amplify the impacts of mesoplastics. There is relatively little research on plastic pollution in crude salt (1.22%) and the distribution of plastics in mangrove ecosystems (1.22%) [30]. However, mangrove ecosystems are considered to be one of the important coastal zones that act as a sink for plastics [27,31,32].

The density and mass of mesoplastics in coastal ecosystems vary greatly from one ecosystem to another. Baseline data on mesoplastics in the Philippines showed more mesoplastics on urban beaches than on non-urban beaches [13]. Fragmented, medium-sized plastics of various colors are widespread along the coast of northeastern Tunisia, and most of them are composed of high-density polyethylene (HDPE) polymer [33]. Compared to the deep waters of the Bay of Biscay, the coastal waters have higher levels of meso- and microplastic pollution [34]. The enclosed Baltic Sea (0.16 pieces/kg) contains more meso- and microplastics than the semi-enclosed Gulf of Riga (0.10 pieces/kg) [35]; the mass of mesoplastics is twice that of microplastics, which are primarily pellets and fragments [36], and the density of mesoplastics in Principe in the Gulf of Guinea was found to range from 0 to 6.78 items m^−2^ [37].

Soils and agricultural lands are also affected by mesoplastic pollution. The soils of the Liaohe River basin in China are contaminated with mesoplastics primarily due to industrial and agricultural activities, population density, and sewage treatment plants [38]. A study conducted on soils from residential areas near the Tehran landfill in Iran shows that the most prevalent mesoplastic particles were film-shaped and made up of LDPE, and the potential ecological risk across all sampling points was low [39]. Additionally, the Galuga Landfill in Indonesia generates and releases an average of 618,240 mesoplastics, and its amount can increase nine-fold for mesoplastics after tan input of leachate drain [40]. Studies on mesoplastic pollution in agricultural lands across the globe determined that macroplastics are relatively abundant compared to meso and microplastics in the horticultural soils of Argentina [41], mesoplastics (2.22 items/kg) are more prevalent than microplastics (0.83 items/kg) on the small-scale agricultural areas of Iligan City, Philippines [42], and the abundance of mesoplastics varies across different regions in China [21,22,43]. Also, water-irrigated fields have a greater number of microplastics. The amount of mesoplastics also varies across different types of water irrigations, but overall, fibers are the most common type of plastics contaminating farmlands [44]. The sources of pollution include the usage of pesticides in plastic bottles and packaging, sewage discharge, irrigation, and foam materials used in packaging and decoration [38]. The use of plastics in agricultural activities has increased efficiency and harvest. However, improper management of its waste can cause problems [45]. Its negative impact on soil health includes infiltration into plant roots and hindrance of the absorption of water and nutrients. It can also lead to toxicity, which can threaten agricultural sustainability [45].

### 3.3. Phenomena Affecting the Prevalence of Mesoplastics

The prevalence of plastic pollution in an area can be influenced by various factors, including human-induced activities and natural phenomena (Table 2). Over recent years, human activities have been considered to be the primary source of plastic pollution because of the extensive production and consumption of plastics in the agriculture, industry, transportation, and packaging sectors, which has intensified the challenge of proper plastic waste disposal and environmental contamination [2,46]. Although recycling is considered the most eco-friendly solution for the increased plastic waste, many countries in the developing world, like Bangladesh, still have limited recycling capability, with only 9.2% of plastic waste recycled in 2014 [47]. Improper disposal practices, lack of waste management infrastructures, lack of public awareness, the high cost of recycling, and the limitation in technology significantly contribute to the leakage and accumulation of plastics in the natural environments [47,48,49]. Moreover, the plastic debris degraded at landfill sites can enter aquatic environments through leachate drains, outlet channels, and pond leaks. High micro- and mesoplastic concentrations from leachate ponds in river outlets significantly increase water pollution [40].

Aside from human activities, storms, cyclones, and floods are significant natural disasters that contribute to transferring plastic waste to the aquatic environment and vice versa [58]. For example, storms can quickly transport significant amounts of plastics from coastal areas to open waters [58]. In Sagami Bay, Japan, the concentration and mass of micro-mesoplastics dramatically surged one day after typhoon Faxai hit (i.e., the particle number increased by two orders of magnitude and an increase of 1300 in mass) but returned to their pre-typhoon levels within two days [58]. Another factor that affects mesoplastics’ prevalence is season variability [18,55,56,57]. For example, the number of microplastics and mesoplastics in the waters of Jiaozhou Bay varied throughout the year, peaking in May due to the heavy rainfall accompanied by strong winds [18]. The majority of the collected plastic debris was made up of polypropylene and polyethylene polymers and was mainly in the shape of fragments (55%) and fibers (29%) [18].

The direction and frequency of Stokes drift can also affect the number of mesoplastics on the sea surface [52,53]. Moreover, the beach location from where the plastic debris is being collected and the presence of a community near the coast can affect mesoplastic pollution [54]. Aside from the residential community nearby, industrial activities can also increase the presence of mesoplastics [55].

### 3.4. Types of Mesoplastics Based on Shape, Color, and Polymer Composition

**Figure 4:**
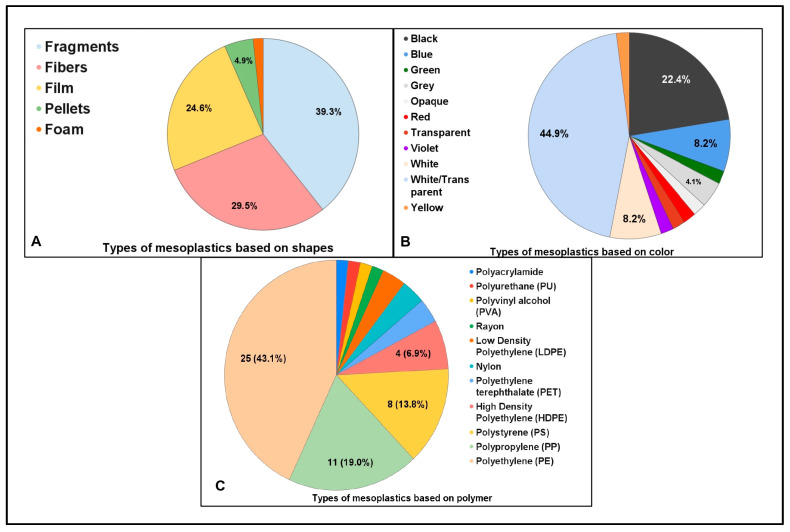
(**A**) Percentage distribution of mesoplastic types based on shape consolidated from published studies (n = 61). (**B**) Percentage distribution of the mesoplastic types-based colors consolidated from published studies (n = 49). (**C**) Percentage distribution of the mesoplastic polymer type consolidated from published studies (n = 58).

Mesoplastics can be differentiated by their shapes (Figure 4A), which depend on how they disintegrate from larger plastics. The most abundant plastic shapes identified by the published articles (n = 61) were listed, and the frequency of each shape type was recorded. Most studies (24) determined that the most abundant type of mesoplastics based on shape are fragments (39.3%); however, identifying the source of contamination of these plastic fragments, especially the secondary fragments, can be challenging since it is difficult to link them to specific applications or point source areas [59]. This is followed by fibers, with 29.5% (18 studies), films with 24.6% (15 studies), and pellets with 4.9% (3 studies). Plastics in the shape of fibers are commonly found in soils that have undergone sewage sludge treatment or those irrigated with contaminated water, while fragments are often the result of long-term environmental exposure [60]. Plastics with shapes that differ significantly from soil particles, such as fibers and films, can impact soil properties more strongly compared to spherical-shaped plastics that resemble natural soil particles [60]. As for pellets, their low concentrations could be due to poor long-distance dispersers, as shown by limited dispersal across the Sea of Japan [61]. In South Africa, mesoplastic pellets, especially industrial pellets, are concentrated near urban sources, meaning their proximity to industrial factories is a stronger predictor of debris abundance than population density [62]. Figure 4A highlights the prevalence of different plastic shapes, providing insight into potential sources and environmental impacts of mesoplastic pollution.

The majority of the research articles (n = 49) determined that the most abundant mesoplastics collected were white/transparent (44.9%) in color (Figure 4B). This result is unsurprising since white plastics are the most commonly manufactured color [63]. Also, the high proportion of transparent and white items is due to the prevalence of single-use or disposable plastics in these colors, as well as bleaching from environmental factors, particularly ultraviolet radiation [64]. This is followed by black (22.4%), white and blue (8.2%), and grey (4.1%). This indicates that these colors make up a substantial majority of the mesoplastics’ characteristics. In terms of recycling, there are challenges that can be encountered with handling black plastics, mainly due to their low reflectance. A study by the authors of [65] highlighted the usefulness of the Midwave Infrared (MWIR) spectral approach for better identification of black plastics, effectively addressing the limitations of Near Infrared (NIR) methods caused by the low reflectance of dark materials. The color of plastics can affect the probability of ingestion of plastics by different organisms. As such, the color of plastic debris could have an ecological relevance since it has been shown that fishes and invertebrates commonly ingest plastic particles that are white, transparent, and blue in color [29,66].

Figure 4C shows the most abundant polymer types of mesoplastics in published articles (n = 58). Polyethylene (PE) plastics are the most prevalent, comprising 43.1% of the total, indicating their significant dominance during the study. This polymer is highly resistant to biodegradation due to it being water insoluble, having a hydrophobic carbon backbone, having a high crystallinity, and also due to its substantial molecular weight [67]. Also, it only shows partial degradation even after being buried in moist soil for 12–32 years [67]. The next most common type of polymer identified is polypropylene (PP), with 18.97%, followed by polystyrene (PS) with 13.79%, and high-density polyethylene (HDPE) with 6.9%. Polypropylene is synthesized catalytically from propylene, and it is notable for its excellent chemical resistance, versatility in processing, and resistance to high temperatures [68]. With this, manufacturing companies take advantage of this polymer to produce goods such as trays, funnels, pails, bottles, instrument jars commonly used in clinical settings, and other items that undergo frequent sterilization [68]. Polystyrene is a durable and low-cost plastic polymer that is commonly designed to create goods that are supposed to be used for a very short period of time, such as disposable plastics. Polystyrene is mostly used in disposable cups, spoons, forks, and Styrofoam [69]. In addition, HDPE is one of the most common classifications of polyethylene, which differs from LDPE based on the polymerization of its ethylene and its polymer chain of up to 1,000,000 carbon units [70]. This polymer is used for milk bottles, freezer bags, distilled water, carrier bags, ice cream containers, shampoo bottles, detergent bottles, liquid soap bottles, lotion bottles, and bleach bottles. Polyacrylamide is the least common type of mesoplastic, showing its rarity in this dataset. Overall, the chart highlights the prevalence of polyethylene and polypropylene, with other materials having considerably smaller shares.

The degradation of larger plastic debris in the environment generally begins with photodegradation, where ultraviolet (UV) light from the sun triggers the incorporation of oxygen atoms into the polymer, causing it to fragment into much smaller plastic particles such as microplastics. Further chemical degradation commonly involves oxidation and hydrolysis, in which chemical plastic molecule bonds are cleaved upon exposure to light, oxygen, or water and transform into smaller molecules such as aldehydes, ketones, and carboxylic acids [71]. Other mechanisms include environmental factors like heat and mechanical stress that degrade plastics into smaller pieces [71]. In biological degradation, plastics are metabolized by some microorganisms and fungi, which degrade them into smaller biodegradable products like fatty acids, alcohols, and other organic compounds [6,72]. However, due to the nature of plastic, its degradation can be a very slow process that can take over 50 years. In marine waters, plastic degradation can be much slower due to lower temperatures, reduced oxygen availability, and the limited hydrolysis rates of most polymers [26,73]. Plastic degradation results in the release of a number of chemicals, including benzene, styrene, phthalates, and other polycyclic aromatic hydrocarbons (PAHs), all of which have proven to be environmentally and biologically toxic [6].

Plastics can originate from industrial products and packaging materials and eventually break into smaller pieces in natural environments. They enter ecosystems through improper waste management, riverine transport, and coastal accumulation [26]. The presence and concentration of plastics in the aquatic ecosystem are affected by their composition, density, porosity, and shape. These characteristics can help determine whether they can float in surface water or settle in sediments [51,74]. Beaches are considered significant reservoirs of plastic debris across polymer types, with their accumulation being influenced by wind speed, direction, and wave action, favoring coastal deposition during certain periods. The presence of mesoplastics and microplastics in bottom sediments is also attributed to weathering and deposition processes over time [51].

### 3.5. Association of Mesoplastics with Macro-, Micro- and Nanoplastics

**Figure 5:**
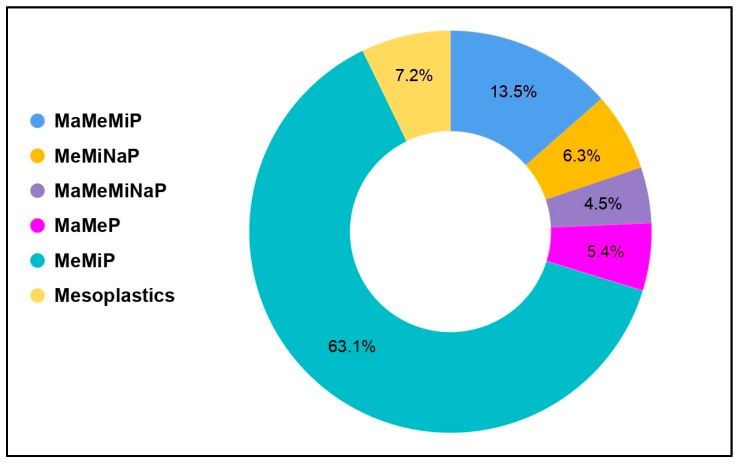
Association of mesoplastics with macro, micro, and nanoplastics in terms of the number of published papers. Note: macro, meso, microplastics = MaMeMiP; meso, micro, nanoplastics = MeMiNaP; macro, meso, micro, nanoplastics = MaMeMiNaP; meso-macroplastics = MaMeP; meso-microplastics = MeMiP; meso-nanoplastics = MeNaP.

The number of studies that associated mesoplastics with other plastic sizes, namely macroplastics, microplastics, and nanoplastics, is illustrated in Figure 5. This relationship refers to the proportion of published articles (n = 111) referencing the combination of mesoplastics with other types of plastics. Most of the studies conducted globally focused on the collection of meso- and microplastics (MeMiP), comprising 63.1% of the total. This suggests that researchers are interested in the interaction and co-prevalence of these plastics in certain ecosystems, recognizing that plastic pollution exists as a continuum rather than as isolated size categories. This is followed by the combination of macro-, meso-, and microplastics (MaMeMiP) (13.5%), studies on mesoplastics alone (8.4%), and meso-, micro-, and nanoplastics (MeMiNaP) (6.3%). The studies on MaMeMiP and MeMiNaP can indicate the efforts to uncover mesoplastic interconnections with two or more plastic size categories, showing how mesoplastics interact within a broader spectrum of plastic pollution types. Small plastic fragments, 5 mm to 50 mm in size, can act as a bridge between macro-, micro-, and nanoplastics; therefore, investigation of this plastic category can address the gaps in management and the development of policies [75]. In the study conducted in the Japanese rivers, there was a significant increase in the concentrations of microplastics and mesoplastics, along with an increase in microplastics. Due to this, it was concluded that the combined concentration of the meso-microplastics should be monitored to prevent an underestimation of plastic contamination [76]. It is also important to have a standardized method and uniform protocols when it comes to the sizing of plastics and the collection of plastic samples to ensure that there can be valuable comparisons with other studies [77]. Furthermore, studies that solely focus on mesoplastics show that moderate attention is given exclusively to mesoplastics, without direct association with other plastic sizes. Among the interactions of all the plastic sizes, no direct interconnections were made between the meso- and nanoplastics (MeNaP). The diverse distribution of proportions across these plastic size combinations reflects a predominantly concentrated research effort on microplastic and mesoplastic interactions. This distribution of research efforts suggests a potential gap in understanding the full range of interactions between different plastic sizes, particularly for complex combinations involving all size categories.

### 3.6. Analytical Methods Used in Mesoplastic Studies

#### 3.6.1. Collection Method

There are several methods for collecting mesoplastics (Table 3). The most common technique in marine surface water is the manta trawl, accounting for 42.9% of the studies reviewed. This is followed by the Neuston net, utilized in 21.4% of the cases; other types of nets were used in 14.3% of the studies. The plankton net is the predominant tool for mesoplastic collection from freshwater surface water, used in 50% of the studies. The predominance of these surface water methods for collection reflects their adaptability and efficiency in capturing small particles suspended in water bodies. As for the marine and freshwater sediments (i.e., including the beach), the most common method used was the transect line method, with 90% and 87.5%, respectively. This result demonstrates its effectiveness in systematically sampling mesoplastics across sediment layers. When studying mesoplastics in biotic samples, methods vary by ecosystem. For the marine biota, studies utilized both the direct catch method (50%) and the purchasing of samples from available markets (50%), while the freshwater biota was mostly directly caught (100%). In the terrestrial and agricultural ecosystem, the most common method used is the direct collection of soil samples from the area (80%), while for the biotic factor, all of them were directly caught. These results reflect the straightforward nature of sampling in these settings. Overall, the choice of method reflects the environmental conditions and the specific objectives of the study, with the primary aim being to ensure an efficient and representative collection of mesoplastic samples.

#### 3.6.2. Digestion

**Figure 6:**
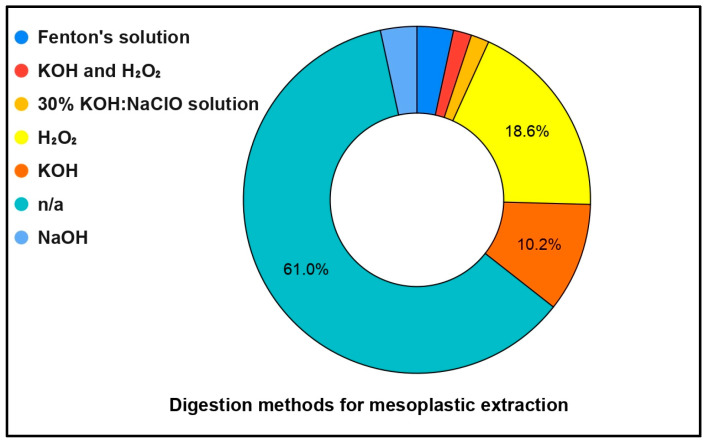
Digestion methods utilized by the authors for mesoplastic analysis.

Figure 6 illustrates the various digestion methods used for mesoplastic extraction. Majority of the studies did not specify the digestion method used, or they did not use any digestion method at all (61%). Adding H_2_O_2_ is the most frequently used method, accounting for 18.6% of the cases, demonstrating its ease and effectiveness in breaking down organic matter. For this method, H_2_O_2_ is added to the supernatants to facilitate the degradation of organic matter. The mixture is then processed under controlled conditions and filtered through fine nylon mesh using a vacuum pump to isolate the desired materials [21]. Following H_2_O_2_, KOH is the next most utilized (10.2% of studies). Other methods, such as Fenton’s solution, KOH with H_2_O_2_, and a 30% KOH:NaClO solution, are applied less frequently. The graph shows the authors’ strong preference for simpler and widely available chemical digestion techniques in mesoplastic research.

#### 3.6.3. Density Separation

**Figure 7:**
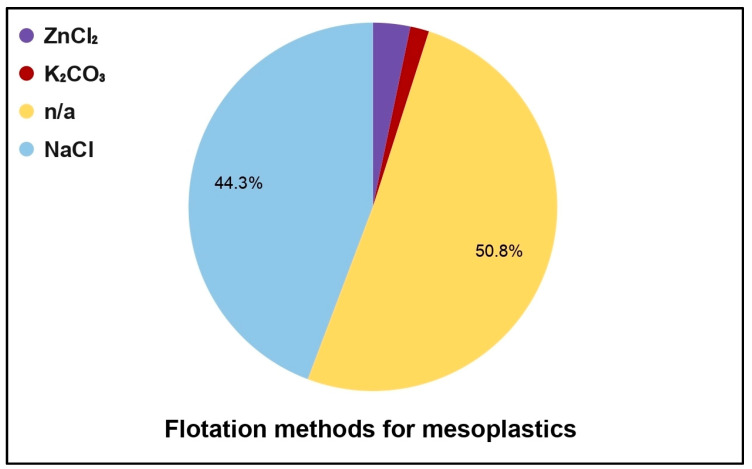
Flotation methods utilized by the authors for mesoplastic density separation.

The figure illustrates the density separation methods used for extracting mesoplastics (Figure 7). Most of the reviewed papers did not state the method of mesoplastic analysis or did not conduct any digestion of mesoplastics (50.8% of the cases). This is closely followed by using NaCl solution, representing 44.3% of the studies. In general, utilizing NaCl solution for plastic floatation involves adding the solution to a vial with the samples. Next, the mixture is stirred for an extended period to ensure effective removal of the target materials and to separate the plastics from unwanted materials [78]. The remaining floatation methods, ZnCl_2_ and K_2_CO_3_, were significantly less utilized, being reported in only a few studies. This distribution highlights a strong preference for simple or unspecified methods, while more specialized solutions are less commonly employed in mesoplastic research.

#### 3.6.4. Identification

Based on the papers reviewed for this article, all authors utilized the Fourier Transform Infrared Spectrometer (FTIR). FTIR is a practical technique popular for its ability to identify and characterize plastics using their polymers by analyzing their distinct molecular vibrations and infrared absorption spectra. The FTIR spectrometer is commonly used to identify various types of plastic polymers. One type of FTIR technique is the Attenuated Total Reflectance-Fourier Transform Infrared (ATR-FTIR). An ATR-FTIR spectrometer is commonly used to determine the molecular composition of plastic polymers by analyzing their infrared absorption spectra. This tool is highly effective for identifying polyethylene, polypropylene, and polystyrene polymers, which makes it very useful for recycling efforts [79].

#### 3.6.5. Units of Measurement Used

**Figure 8:**
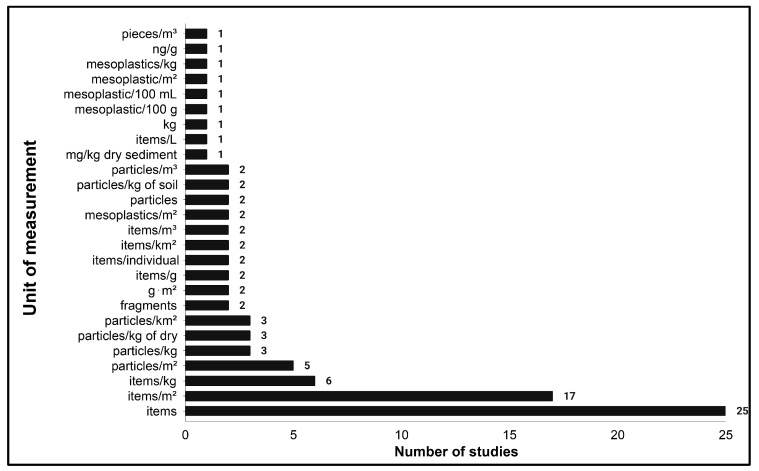
Trends of units of measurements of mesoplastic concentrations.

Figure 8 shows the different units reported across mesoplastics studies, indicating a lack of standardized measurement tools and indices for data reporting. Published studies (n = 91) were categorized based on the unit of measurement that researchers used to determine the presence of mesoplastics. The most commonly used unit of measurement is “items”, with 25 studies using this unit. This is significantly more abundant than other units. This unit’s high number of users reflects a preference for simple item counts or frequency over complex and derived units, which could be due to its ease of implementation during field sampling. Following this, “items/m^2^”, “items/kg” and “particles/m^2^” were used in 17, 6, and 5 studies, respectively, indicating a focus on spatial density metrics in many studies. Other units like “particles/kg”, “items/gram”, and “mg/kg dry sediment” reflect weight-based or volumetric quantifications of mesoplastic pollution. Using these units can provide insights into the relative mass of plastics in a given substrate, which may be critical for assessing environmental impacts. Some less commonly used units, such as “mesoplastics/100 mL”, “ng/g”, and “kg”, suggest niche approaches used for specific research contexts or environmental matrices. Furthermore, volumetric units like “particles/m^3^” are used much less despite their potential relevance in aquatic systems. Specialized units of measurement, such as “items per individual”, are used for assessing the biological impacts of mesoplastics. This metric is particularly useful for studying the ingestion of mesoplastics by organisms, helping to quantify the extent of exposure and potential harm.

Using various units demonstrates the multidimensional nature of plastic pollution research, targeting various compartments such as water, soil, and sediment. However, this variability may pose challenges for data comparability across studies, especially in conducting systematic reviews and meta-analyses and synthesizing findings. Overall, the figure emphasizes the variety of units of measurements used to determine the concentrations of mesoplastics. While using “items” as a unit shows some concurrence, the large variety of measurements highlights the ongoing need for methodological harmonization to have better comparability and reliability in mesoplastic pollution and plastic pollution studies [80]. There should be a standardized/universal metric system for investigating mesoplastic concentration and overall plastic concentration so that researchers can create a more cohesive understanding of pollution and policymakers can implement policies to tackle plastic pollution based on existing studies [80]. However, flexibility should also be retained to address specific ecological, geographical, and methodological needs.

### 3.7. Index Tools That Are Used to Determine Mesoplastic Contamination

Various indices are used to assess the impact of the prevalence of mesoplastics and plastics in general. A few of these risk indices include the polymer hazard index (PHI), pollution load index, potential contamination index (PCI), ecological risk index, carbonyl index, pellets pollution index (PPI), clean-coast index (CCI), and beach cleanliness index (BCI) (Table 4).

In the landfill soils of Tehran, located in Iran, the calculated polymer hazard index determined that the hazard levels across sampling points vary, with levels III-IV for microplastics and levels II-IV for mesoplastics, while the pollution load index results showed lower hazard levels for both micro and mesoplastics [90]. On the other hand, the ecological risk index suggests a minor to extreme risk of microplastics and a minor risk of mesoplastics, offering baseline data on MP contamination in Tehran landfill soil to support risk-reduction policy decisions [90]. The polymer risk index-based (PHI) hazard grading technique of meso-microplastics in six common fishes from the Bay of Bengal Coast indicated that the plastic contamination risk in studied fish species varied widely, with PU posing the highest health hazard (Category IV), while PE, PS, and PA showed moderate risks (Category II), and PP and PET posed minor risks. However, these particles can impact other marine life through food web transfer [78]. Also, the contamination factor (CF) and pollution load index (PLI > 1) reveal that fish organs were moderately contaminated with plastics (1 < CF < 3) and that the fish were polluted due to plastic debris accumulation, respectively [78]. A PLI value greater than one indicates high contamination/pollution, while values at or below one imply low contamination. Therefore, it is useful in ecological risk assessments [82,83]. Similarly, the Rivera Beach and Golden Bay of Malta indicate a medium (1.021) and high (1.118) PHI, respectively. The relatively medium environmental and health risk of Rivera Beach is due to the dominance of lower-hazard-score plastics, such as PP and PE, while the higher PHI of Golden Beach is due to the presence of higher-hazard-score plastics, such as PVC, PET, and PS [91]. In Algeria, the pellets pollution index (PPI) results categorized the Skikda beaches into a range of pollution levels ranging from very high, as shown by Kef Fatma, to very low, as shown by Grande Plage, with an average PPI of 3.45, indicating very heavy pollution overall. Compared to the west coast of Algeria, which is classified as moderately polluted, Skikda shows evidence of greater plastic accumulation [36]. Further, the calculated PCIs for all beaches on the Gulf of Guinea coast were less than one, which indicates that there is low metal contamination in the plastics collected from the sediments in the area [92]. In high-latitude nature reserves in Northern China, 32 sites were shown to be contaminated by meso-microplastics, most likely from domestic sewage and aquaculture [93]. However, even though there was an abundance of rayon, polyester, polystyrene, and polyethylene terephthalate, the risk index and the pollution load index still demonstrated a low risk of micro- and mesoplastics in surface water and sediments in the area [93]. In a study conducted in Istanbul (Turkey), the carbonyl index (CI) indicates that 35.7% of the plastic samples were at a low oxidation level, 28.6% at a medium oxidation level, and 35.7% at a high oxidation level, with the lowest value of CI at 0.037 and the highest value at 1.859 [14]. A high level of CI means that the plastic spent too much time in the environment and was exposed to photooxidation caused by UV light [14]. Based on the CCI conducted on various beaches, all the beaches sampled in the Tuticorin district in India are highly polluted (CCI >20), especially the areas with high fishing activities [94]; beaches around Northern Ambon Island have moderate levels of plastic pollution [95]; and, the beaches in Istanbul, Turkey, are considered extremely dirty [14]. A beach cleanliness index conducted for selected beaches on the Northern Strait of Malacca determined that the sites have a BCI less than zero, which is not ideal because of the lack of waste bins and insufficient frequency of beach clean-ups in the area [26].

Of the 15 published studies reporting specific risk and pollution indices, the polymer hazard index has been reported in the highest number of studies (n = 4), followed by the pollution load index (n = 3) and the clean coastal index (n = 3). Thus, it can be concluded that determining the polymer composition of plastics is essential for identifying the wide array of plastic additives leaching from polymers, which can give a comprehensive understanding of the key compounds contributing to the toxicity of plastic products [96]. Other indices, including the potential contamination index, ecological risk index, pellets pollution index, carbonyl index, and beach cleanliness index, were only reported in one published study each, indicating limited use of these indices for plastic contamination determination. It can be deduced that research concentrates on certain indices while others receive minimal attention. Assessment of plastic and mesoplastic pollution using different indices is recognized as essential for determining exposure to pollutants [97]. These indices effectively determine the quality of the environment in a clear and accessible manner by quantifying the presence of various pollutants, allowing for direct assessment of environmental conditions [97]. These indices can provide baseline data that will enable the correct application of risk management strategies to various ecosystems, from terrestrial to aquatic ecosystems. In addition, the results of these indices can assist in the development of pollution reduction measures and promote consistent, comparable environmental quality assessments across diverse regions. Lastly, these indices emphasize the urgent need for sustainable waste management and proactive environmental protection measures worldwide.

### 3.8. Mesoplastics as Vectors of Inorganic and Organic Chemicals

Plastic debris can serve as a transport vector for toxic chemicals, organic pollutants, and heavy metals, adsorbing these substances from the surrounding environment, especially plastics found in aquatic ecosystems [98]. In the water columns and sediments, mesoplastics can adsorb the chemical pollutants that they come into contact with [99]. The exposed mesoplastics have higher amounts of metal concentrations compared to new mesoplastics with the same polymer composition [99]. Other heavy metals are also found to settle on the surfaces of mesoplastic debris collected from different aquatic ecosystems, such as those from the Coast of Peru, which contains Cu and Pb [50]; those from the Gulf of Guinea, with Al, Fe, and Zn being present at the highest concentrations [92] and, plastic debris from Malta Beaches containing the potentially harmful metals Cd, Co, Cr, Cu, Mn, Ni, Pb, Zn, and Fe [91]. In the North Atlantic subtropical gyre, the collected plastic wastes had significantly higher concentrations of titanium, vanadium, nickel, strontium, arsenic, and zinc compared to new packaging materials, with arsenic levels particularly significant due to its restricted use in formulations. Additionally, barium concentrations were generally higher in plastic wastes due to their use in pigments [100]. Among the endocrine-disrupting chemicals (EDCs) found on the sampled plastic debris in the study by [101], estrogen was the most dominant and was either absorbed from the environment or originated from the manufacturing processes that the plastics underwent. Ref. [102] also determined the presence of 52 chemicals in plastics in the forms of antioxidants, phthalates, ultraviolet stabilizers, hindered amine light stabilizers, and flame retardants, which conform with those in the study by [103], who also reported organophosphate esters and phthalates containing plastics. Mesoplastics from the beaches of Malta also indicated the capacity of other materials to pollute plastics, such as persistent bio-accumulative and toxic chemicals, including polychlorinated biphenyls (PCBs), polycyclic aromatic hydrocarbons (PAHs), organochlorine, and heavy metals [56]. Similarly, [104] also observed chlorinated polycyclic aromatic hydrocarbons (ClPAHs) and brominated polycyclic aromatic hydrocarbons (BrPAHs) on mesoplastics collected from the coasts of Sri Lanka and Japan. In the southeastern Baltic Sea are significant amounts of paraffin (up to 3.8 kg/km^2^) and organic matter that have been linked to increased plastic particle concentrations on the surface layer, which highlights that shipment activities were the main cause of plastic pollution in the area [105]. In addition, other toxic chemicals, heavy metals, and organic matter were found to inhibit plastic debris, especially those collected from aquatic ecosystems (Table 5).

Mesoplastics’ larger size compared to microplastics provides a greater absolute surface area for pollutant adsorption. However, microplastics generally possess a higher specific surface area, which is the relative total surface area volume, enhancing their capacity to adsorb pollutants [107]. Thus, smaller microplastics (<0.9 mm) have greater metal adsorption capacity compared to larger microplastics (0.9–5 mm), highlighting the influence of particle size on adsorption efficiency [107]. However, mesoplastics are effective adsorbents of toxic chemicals under certain conditions. For example, [106] determined that the sorption ability of nitrobenzene on low-density polyethylene (LDPE) mesoplastics was higher compared to that of LDPE microplastics. Also, the sorption ability of naphthalene on polyvinyl chloride (PVC) mesoplastics exceeded that of PVC microplastics. This is because of the sorption capacity of the functional groups present on its surface [106]. Furthermore, environmental aging processes can increase the surface roughness and porosity of both mesoplastics and microplastics, potentially enhancing their adsorption capacities over time [108]. Therefore, while microplastics may inherently possess higher adsorption capacities due to their smaller particle size, the actual adsorption potential of both mesoplastics and microplastics is influenced by a combination of factors, including particle size, surface characteristics, porosity, and environmental conditions.

### 3.9. Mesoplastics Affecting Organisms

**Figure 9:**
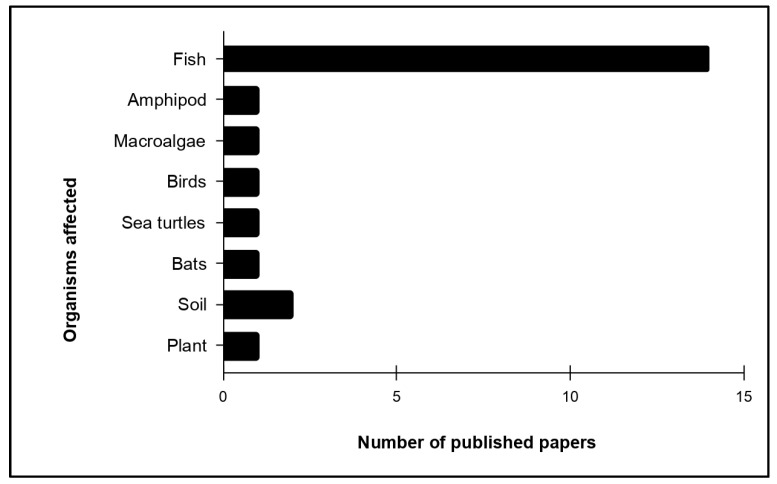
Number of published articles that tackle the impact of mesoplastic pollution on certain organisms.

Mesoplastic pollution has been found in various organisms (Figure 9). Among these organisms, fishes (n = 14) from both marine and freshwater ecosystems were the most investigated, which is not surprising since the aquatic ecosystem is also the most studied. Amphipods, macroalgae, birds, sea turtles, bats, and plants have one published article each, and soil microorganisms have two published studies, indicating a relatively low research focus on these organisms.

Aside from the direct impact of mesoplastic pollution on the environment, mesoplastics can also harm organisms indirectly. Plastics of different sizes, primarily microplastics and mesoplastics, were found to affect small and large-sized fishes. All twenty-one marine fishes and six freshwater fishes collected from the waters of China were determined to have ingested meso and microplastics, with fibers being the most abundant [12]. Easter Island flying fish (*Cheilopogon rapanouiensis*) and yellowfin tuna (*Thunnus albacares*) from Rapa Nui (Easter Island) also exhibited plastic ingestion; however, it was mostly dominated by microplastics rather than larger plastic debris [109]. Moreover, it was emphasized that ingesting these small plastic debris was not a direct threat to the large yellowfin tuna [109]. In the Bay of Bengal, meso- and microplastics, mostly polyethylene, polyamide, and polyester, were also present in the gastrointestinal tract of some commercially important fish [110]. Coral reef fishes and mangrove fishes in Isla Grande, Colombia, were also found to have ingested plastic debris [111]. The fish in mangroves were determined to have ingested more meso-microplastics because these areas experience low water currents that may cause plastic debris to store the plastic for longer, giving the fishes more opportunity to prey on this debris [111]. Mesoplastics were also detected in the guts of fin whales feeding in the Pelagos sanctuary of the Mediterranean marine protected area [112], reef fish across the east coast of Australia [113], teleost fish in the Gulf of California [114], pelagic and demersal fish in the Western Mediterranean Sea [115], and the common fishes in Noakhali-Feni and Chittagong coastal zone of the Bay of Bengal [78].

In addition to marine fish, freshwater fish can also be impacted by mesoplastic pollution. Mesoplastics have also been found in the guts and gills of an economically important fish commonly caught in Ambattur, Korattur, and Madhavaram Lake, the Oreochromis niloticus [116]. Mesoplastics were also present in canned sardines sold in Australian and Malaysian markets; however, it should be noted that these plastics contained no toxic compounds upon examination [117]. The low level of plastic particles found in the sardines can indicate a low health risk when consumed by humans [117]. The plastics accumulated in various parts of the digestive tract of fishes can gather in the gastrointestinal cavities, hindering foraging activity, and micro and nanoplastics degraded from mesoplastics can infiltrate tissues and disrupt chemical signaling [111]. Furthermore, plastics can remain in the fish’s intestines for weeks, leading to malnutrition, physical deterioration, and eventual starvation [111]. Macroalgae were also contaminated by mesoplastics [118]. Five species of macroalgae found on the coast of China, namely *Gracilaria lemaneiformis*, *Chondrus ocellatus*, *Ulva lactuca*, *Ulva prolifera*, and *Saccharina japonica* were found to be polluted by plastics, mostly micro- and mesoplastics [118]. The macroalgae could accumulate these plastics through entanglement, adherence, wrapping, embedment, and entrapment by epibionts [118].

Coastal and marine bird species can also ingest mesoplastics through their prey that ingested plastic particles; additionally, these plastics may resemble their usual foods [1]. Ingested plastics can harm birds by obstructing food passage, causing digestive issues, and reducing food consumption, leading to starvation and reproductive delays, resulting in internal injuries and contributing to mortality and population decline [1]. Loggerhead and green sea turtles found ashore on Florida’s Atlantic coast (USA) have plastic particles in their guts [119]. Their potential impacts on these turtles include intestinal impaction, gut perforation, and gut blockages [119]. Lastly, neotropical bats from the Brazilian Amazon were recorded to have inhaled and ingested meso-microplastics [120]. The contamination of the digestive system of bats occurs through bioaccumulation and biomagnification through their consumption of contaminated food or water. In contrast, the respiratory system is contaminated by inhaling plastic particles suspended in the air [120]. The impact of plastic pollution can also be seen in microorganisms. For example, the presence of plastics of different types and sizes in soil can affect the normal biological process of waste degradation, and it can also impact the process of vermicomposting since this organic matter can be reintroduced in agriculture as fertilizer [121]. It was discovered that the presence of plastics negatively affected the survival of Eisenia fetida by ten percent and also reduced its weight [121]. Also, the growth of soil invertebrate communities, especially the Collembola, is affected by mesoplastic contamination through the reduction of richness and abundance after its exposure [122].

The plant community is also affected by the presence of mesoplastics. In [122], the authors investigated the effects of conventional polyethylene, polypropylene, and alternative (biodegradable polyethylene and compostable polylactic acid) mesoplastic films on plant performance. They discovered that mesoplastics affected 11-week-old spring barley (*Hordeum vulgare*) through reduced biomass, seed yield, and chlorophyll content, alongside increased oxidative stress, regardless of plastic type.

Overall, the harmful impact of mesoplastics on organisms is mainly due to ingestion. Ingested plastic debris can pose significant threats to organisms, including intestinal blockages that may cause indigestion due to the limited stomach capacity of organisms, inhibiting gastric enzyme secretion, reducing feeding stimuli, decreasing steroid hormone levels, delaying ovulation, and impairing reproduction [113,123]. Also, ingesting these materials will likely result in net energy loss [113].

However, the large gap in the number of research published on the impact of mesoplastics on organisms suggests that it is heavily concentrated on fish, while other organisms receive minimal attention. The lack of studies on other organisms suggests gaps in understanding the total environmental impact of mesoplastic pollution. There is a need for more balanced research efforts across diverse organisms to gain a much better understanding of the impact of mesoplastic contamination in the fauna community. Furthermore, a more holistic approach could determine hidden risks and allow for the development of more effective mitigation strategies for the safety of the fauna community. In addition, investigating the possible long-term effects of mesoplastics on animals’ health, reproduction, and biodiversity is crucial for understanding the broader ecological consequences.

### 3.10. Mesoplastics as a Habitat of Microorganisms

Biotic organisms can also accumulate on plastic debris (Table 6). Invasive species can use microplastics and mesoplastics as floating “rafts” to reach new habitats, with microplastics providing ample surface area for microorganisms to colonize due to their high surface-area-to-volume ratio. The exposure of these plastics to various environmental conditions can enhance this by adding organic matter to plastic surfaces, supporting bacterial growth, diatoms, and plastisphere development, commonly called the formation of biofilm [29]. Harmful algae and pathogens were discovered to accumulate on the plastic debris collected from the Mediterranean coastal lagoons, with an increase in microalgae depending on the season [124]. Diatomophyceae were the most dominant, along with *Alexandrium* spp. and *Vibrio* sp. [124]. A study by [125] also highlighted that microbial biofilm, which can include potentially dangerous bacteria and fungi, quickly colonizes plastics in the environment. This can enhance the risk to human health when people are directly exposed to plastics on beaches or in swimming pools [125]. Mesoplastics collected from the Antarctic Peninsula have shown the presence of diatoms, bacteria, and microalgae on the plastics’ surfaces [126]. Diatoms like *Thalassiosira*, *Synedropsis*, *Chaetoceros*, and *Navicula* were found, with *Synedropsis* notably covering extruded polystyrene foam alongside coccoid and filamentous bacteria on various plastic surfaces [126]. Moreover, [127] determined that the plastics collected from Patos Lagoon and Itajaí Açu estuaries were associated with various organisms, the majority of them being eukaryotes, and the most dominant groups are the diatoms, ciliates, dinoflagellates, radiolarian, and bryozoans. Pathogens, including *Vibrio* sp. and *Alexandrium tamarense*, were detected among these organisms [127]. In the Bermuda Platform, plastic debris was inhabited by various bacteria and was dominated by Alteromonadaceae, Marinomonadaceae, Saccharospirillaceae, Vibrionaceaem, Thalassospiraceae, and Flavobacteriaceae, Hyphomonadaceae, Rhodobacteraceae and Saprospiraceae family [128]. Moreover, the colonizing bacteria varies among the different plastic polymers [128].

Microorganisms can colonize plastic debris depending on its composition, and these microorganisms can include both beneficial and harmful microorganisms, which can alter the ecological balance of the surrounding ecosystem. Various types of plastics can support different microbial communities, as certain compositions of these plastics may provide suitable conditions for specific organisms to prosper and multiply. Additionally, the prolonged existence of plastics in the environment can increase these effects over time, creating hotspots of microbial activity.

### 3.11. Utilization of Various Organisms for Plastic Monitoring and Degradation

Living organisms, both macro and micro, have the capacity to catalyze mesoplastic degradation and aid in monitoring its presence in the environment. In monitoring mesoplastics, [129] investigated the ability of water moss (*Fontinalis antipyretica*) to entrap plastic particles in aquatic ecosystems. This species has a high retention capability and can be utilized for biomonitoring river meso- and microplastics [129]. Plastic degradation processes can be catalyzed through bacterial degradation, using their hydrolase and oxidase enzymes; this method is less likely to have environmental damage [130]. A few other methods that can lessen the use of plastics or anything that can reduce the harmful impact of plastics include the application of nanotechnology in packaging materials, utilization of natural fibers, polylactide (PLA), nano-cellulose instead of plastic polymers, and chemical recycling of plastic wastes [130]. A limited number of bacteria and fungi are known to partially degrade PET plastic particles, with most of them belonging to the Gram-positive phylum Actinobacteria, particularly genera such as *Thermobifida* and *Thermonospora*. The bacterium *Ideonella sakaiensis*, discovered in plastic-contaminated environments, can utilize PET as its sole carbon and energy source, secreting enzymes such as PETase to effectively hydrolyze PET into harmless monomers [131]. In addition, Odonata larvae of *Chironomus* sp. were shown to aid the fragmentation of mesoplastic fibers into microplastics through mechanical comminution with the use of their gizzards, which contain strong chitinous teeth [132]. Following ingestion of these insects, macerated plastic fibers were observed in the feces of several individuals [132]. When it comes to the impact of mesoplastic pollution on soil health, earthworms have been commonly utilized to investigate the effects of plastic pollution on the ability of the soil to maintain its nutrients [133]. It can be deduced that the utilization of different organisms for both plastic monitoring and plastic degradation means that there are innovative and optimistic approaches that can lessen the prevalence of plastic pollution in the environment. The different natural and enhanced abilities of these organisms can help mitigate the impact of plastic waste.

## 4. Conclusions and Future Perspectives

Mesoplastics are significant environmental pollutants affecting aquatic, terrestrial, and agricultural ecosystems, functioning not as separate pollutants but rather as an integral part of the global plastic pollution crisis. Mesoplastics exist within a continuous spectrum of plastic wastes, beginning from larger plastic debris and gradually breaking down into smaller fragments over time. Their presence and durability in the environment highlight the pervasive nature of plastics.

In aquatic ecosystems, they disrupt food chains, reduce biodiversity, and carry harmful chemicals (Figure 9). On land and in agriculture, they degrade soil quality, affect nutrient availability, and contaminate crops. Organisms are harmed through ingestion, entanglement, or inhalation, leading to blockages, reduced feeding capacity, hormonal imbalances, and reproductive issues. These impacts are driven by factors such as human activities, seasonal changes, storms, and floods. Mesoplastics often act as carriers for organic and inorganic compounds, as well as pathogenic microorganisms, highlighting the need for research into their interactions and potential strategies for degradation and remediation. Indices like the polymer hazard index, pollution load index, and others aid in monitoring pollution hotspots and informing mitigation practices. Future research should focus on underexplored ecosystems, regions, and mesoplastic interactions with other pollutants and organisms.

For future studies, researchers can expand their research into ecosystems that are less studied, such as terrestrial ecosystems and agricultural areas. Globally, mesoplastic studies could be conducted in less studied continents and regions to enable policymakers to map the wide impact of plastic contamination. The fate of mesoplastics in various environmental compartments in one area can also be explored. Additionally, investigating the interactions of the mesoplastics with different microorganisms, macroorganisms, and chemical pollutants can help identify various fast degradation and potential remediation strategies. The toxicity of mesoplastics to various organisms, based on the difference in size and polymer type, is worth pursuing. Also, adding risk and pollution indices in mesoplastic studies can help more people understand the need to lessen its prevalence in the environment. Lastly, public awareness campaigns should be conducted, information, education, and communication (IEC) should be distributed in schools, and policies should be reinforced to mitigate mesoplastic and plastic pollution. There should be a focus on reducing plastic use in industries and promoting alternatives that minimize environmental harm.

## Figures and Tables

**Table 1:** Distribution of mesoplastic studies by geographic region.

Region	Number of Studies	Percentage
East Asia	17	15.6
South America	11	10.1
Southern Europe	11	10.1
West Asia	8	7.3
Southeast Asia	7	6.4
North America	6	5.5
South Asia	6	5.5
Southwestern Europe	6	5.5
Central Europe	3	2.8
Northeast Asia	3	2.8
North Africa	2	1.8
Northern Europe	2	1.8
Northwestern Europe	2	1.8
South Africa	2	1.8
Australia	1	0.9
East Africa	1	0.9
Northeastern Africa	1	0.9
Northwest South America	1	0.9
Oceania	1	0.9
West Africa	1	0.9

**Table 2:** Phenomena that affect the prevalence of mesoplastics.

Phenomena	References
Oceanographic factors, including hydrodynamics and Stokes drift terminal velocity, dependent on fragment size and current	[37,50,51,52,53]
weather conditions	[18,51]
Anthropic influence, including community presence, industrial activities, and the occupation of coastal areas	[51,54,55]
Seasonal variability	[18,28,55,56,57]
Sampling stations	[55]
Upwelling/downwelling ocean periods	[55]
Storm	[58]
Beach location	[54]

**Table 3:** List of methods used for mesoplastic sampling.

Type of Environment	Method of Collection	Percentage
Marine surface water	Manta trawl	42.90%
	Neuston net	21.40%
	Net	14.30%
	Pump	7.10%
	Modelling	7.10%
	Water sample collection through the use of buckets	7.10%
Marine beach and sediments	Transect line	90.0%
	Random sampling	5.0%
	Quadrat	5.0%
Marine biota	Purchased	50.0%
	Direct collection	50.0%
Freshwater beach and sediments	Transect line	87.5%
	Non-randomized sampling	12.5%
Freshwater surface water	Stow net vessel	25.0%
	Plankton net	50.0%
	Collection of water sample	25.0%
Freshwater biota	Direct catch	100.0%
Terrestrial biota	Direct catch	100.0%
Soil	Direct collection	80.0%
	Quadrat	20.0%

**Table 4:** Index tools that are used to determine mesoplastic contamination.

Index	Description	Reference
Polymer hazard index (PHI)	A metric designed to assess both the environmental and health risks posed by different types of polymers based on their chemical constituents.	[81]
Pollution load index (PLI)	A tool that is used to assess the presence of heavy metal pollution, such as the presence of heavy metals in plastics.	[82,83]
Potential contamination index (PCI)	A tool that measures ecological risk due to metal concentrations in sediments.	[84]
Ecological risk index (ERI)	Metric used to assess the potential ecological risks of sediments. Takes into account both the environmental impact of various pollutants in an environment and the combined effects of multiple pollutants	[85,86]
Carbonyl index (CI)	It measures the chemical oxidation of polyolefins and generally indicates the degradation of plastic polymers’ mechanical properties.	[87]
Pellets pollution index (PPI)	A standardized method for assessing plastic pellet pollution and the risks it causes on the surface of sandy beaches. Aids in the monitoring of the beach’s environmental quality.	[88]
Clean coast index (CCI)	A pollution index utilized to assess the cleanliness of a beach based on plastic litter data.	[89]
Beach cleanliness index (BCI)	A descriptive statistic that considers the frequency of beach cleaning (BC), availability of waste bins (AB), anthropogenic activities (AA), and natural factors (NF).	[26]

**Table 5:** Organic and inorganic chemical compounds found in mesoplastics.

Chemical Pollutants Identified	Type of Compounds	Reference
Endocrine-disrupting chemicals (EDCs)	Organic	[101]
Antioxidants, phthalates, ultraviolet stabilizers, hindered amine light stabilizers, flame retardants, Irgafos 168	Organic	[102]
Paraffin, organic matter	Organic	[105]
Polycyclic aromatic hydrocarbons (PAHs), Chlorinated polycyclic aromatic hydrocarbons (ClPAHs), Brominated polycyclic aromatic hydrocarbons (BrPAHs)	Organic	[56,104]
Organophosphate esters, Phthalates, and Phthalate alternatives	Organic	[103]
Polychlorinated biphenyls (PCBs), Organochlorine	Organic	[56]
Mg, Pb, Zn, Cu, Fe, Mn, Ti, K, P, Al	Inorganic	[50,56,92]
Lead and cadmium	Inorganic	[99]
Potentially toxic elements (PTEs) (Cd, Co, Cr, Cu, Mn, Ni, Pb, Zn, and Fe)	Inorganic	[91]
Trace metals	Inorganic	[100]
Naphthalene and benzene	Organic	[106]

**Table 6:** Microorganisms found in mesoplastics.

Microorganisms Identified	Study Area	Reference
Diatoms Bacteria Microalgae	Antarctic Peninsula	[126]
Proteobacteria Cyanobacteria Bacteroidetes	Continental Shelf Off the Coast of South Brazil	[127]
*Diatomophyceae* *Alexandrium* spp. *Prorocentrum* sp. *Vibrio* sp.	French Mediterranean Coastal Lagoons	[124]
Microbial biofilms Fecal indicator organisms Pathogens with antimicrobial resistance and thermotolerance	Firth of Forth, Scotland	[125]
*Marinomonas* *Thalassospira* *Vibrio* *Oleibacter* *Alteromonas*	Bermuda Platform, Ferry Reach	[128]

## Data Availability

The data that support the findings of this study are available on request from the corresponding author, H.P.B.
